# Clinical analysis of extralobar pulmonary sequestration with torsion in children: report of 6 cases

**DOI:** 10.1186/s13019-022-01921-8

**Published:** 2022-06-29

**Authors:** Yunxing Ti, Yuanxiang Wang, Junrong Huang, Fengnan Zheng, Qing Zhang

**Affiliations:** Department of Cardiothoracic Surgery, Shenzhen Children’s Hospital, No.7019, Yitian Road, Shenzhen, 518038 Guangdong China

**Keywords:** Extralobar, Pulmonary sequestration, Pedicle torsion, Children

## Abstract

**Background:**

Extralobar pulmonary sequestration is an uncommon congenital pulmonary malformation. Clinically, pedicle torsion of extralobar pulmonary sequestration is extremely rare. Due to inadequate awareness of its atypical presentation and imaging characteristics, clinical diagnosis is very difficult, and it is extremely easy to misdiagnose.

**Case presentation:**

There were 6 children (3 males and 3 females), aged 3–12 years old. The main clinical symptoms of the children were abdominal and chest pain (3 cases), abdominal pain (1 case), chest pain (1 case), and vomiting and abdominal distension (1 case). Two cases were accompanied by fever. Preoperative ultrasound revealed a well-bordered mass with soft-tissue density, accompanied by pleural effusion. On contrast-enhanced computed tomography scans, the mass showed no obvious enhancement. A blood supply was only present in 1 case, and there was no feeding artery shown in the other 5 cases. Extralobar pulmonary sequestration with haemorrhagic infarction was pathologically confirmed. On postoperative days 2–6, the children were discharged uneventfully. There were no complications during the median follow-up of 4 months.

**Conclusions:**

Torsed extralobar pulmonary sequestration usually occurs in childhood or adolescence, with abdominal and/or chest pain as the primary symptoms. Imaging examination shows a well-defined soft-tissue mass without enhancement. The feeding vessel is not clearly displayed in the mass, and extralobar pulmonary sequestration is accompanied by varying amounts of pleural effusion. Video-assisted thoracoscopic surgical resection is associated with excellent prognosis.

## Background

Pulmonary sequestration (PS) is an uncommon congenital pulmonary malformation, accounting for 1.5% of all congenital pulmonary malformations [1]. It is characterized by a nonfunctioning mass of lung tissue that shows no normal communication with the tracheobronchial tree and receives its vascular supply from the systemic circulation [2]. PS is anatomically divided into intralobar pulmonary sequestrations (ILS) and extralobar pulmonary sequestrations (ELS) according to whether there is a complete visceral pleura. ELS comprises 25% of all pulmonary sequestrations [3]. The majority of ELS is located between the lower lobe of the lung and the diaphragm [4–6], and its feeding artery mainly originates from the thoracic aorta, abdominal aorta, or other vessels in the systemic circulation. Venous drainage reaches the right atrium via the azygos vein, hemiazygos vein or vena cava [7]. The overall incidence of ELS is very low, and concurrent pedicle torsion is extremely rare. To date, only 13 paediatric cases have been reported in the English literature. To improve the diagnosis of ELS with pedicle torsion by paediatricians, herein, the clinical data of 6 children with ELS with torsion of the pedicle in our hospital were analysed.

## Case presentation

### Patients’ general information

Six cases comprising 3 males and 3 females were studied, with an age range of 3–12 years and a weight range of 15.4–38.4 kg. The ELS was located on the left side in 2 cases and on the right side in 4 cases. All children were free of comorbidities. The general information of these 6 children is summarized in Table 1.

**Table 1:** The general information of the children

Patient No	Age (y)	Sex	Weight (kg)	Chief complaint	Location	Imaging examination for diagnosis	Preoperative diagnosis	Feeding artery on image	Combined deformity	Pathological diagnosis
1	7	Male	22.4	Vomiting and abdominal distension	Left	Enhanced CT scan and ultrasound	Neurogenic tumour/ELS, intestinal obstruction, pleural effusion	−	−	ELS with haemorrhage, necrosis and myofibroblast proliferation
2	3	Male	15.4	Abdominal pain and fever	Left	Enhanced CT scan and ultrasound	Lung consolidation, pleural effusion	−	−	ELS with haemorrhage and necrosis
3	5	Male	25	Chest pain	Right	Enhanced CT scan	Neurogenic tumour/ELS	−	−	ELS with haemorrhage and necrosis
4	6	Female	22.1	Chest and abdominal pain	Right	Enhanced CT scan and ultrasound	Torsion ELS, pleural effusion	+	−	ELS with haemorrhage, necrosis and myofibroblast proliferation
5	10	Female	34.6	Chest and abdominal pain, fever	Right	Enhanced CT scan and ultrasound	ELS, pleural effusion, severe pneumonia	−	−	ELS with haemorrhage and necrosis
6	12	Female	38.4	Chest and abdominal pain	Right	Enhanced CT scan	Torsion ELS, pleural effusion	−	−	ELS with haemorrhage and necrosis

*y* Year, *kg* kilogramme, *ELS* Extralobar pulmonary sequestration, *CT* Computed tomography

“+” means presence and “−” means absence or unclear diagnosis

### Clinical manifestations

The initial symptoms of the 6 children on admission included abdominal pain and chest pain in three cases, abdominal pain in one case, chest pain in one case, and abdominal distension and vomiting in one case, and two of them had fever. The course of illness ranged from 1 to 10 days (median, 5 days).

### Imaging examinations and diagnosis

**Fig. 1:**
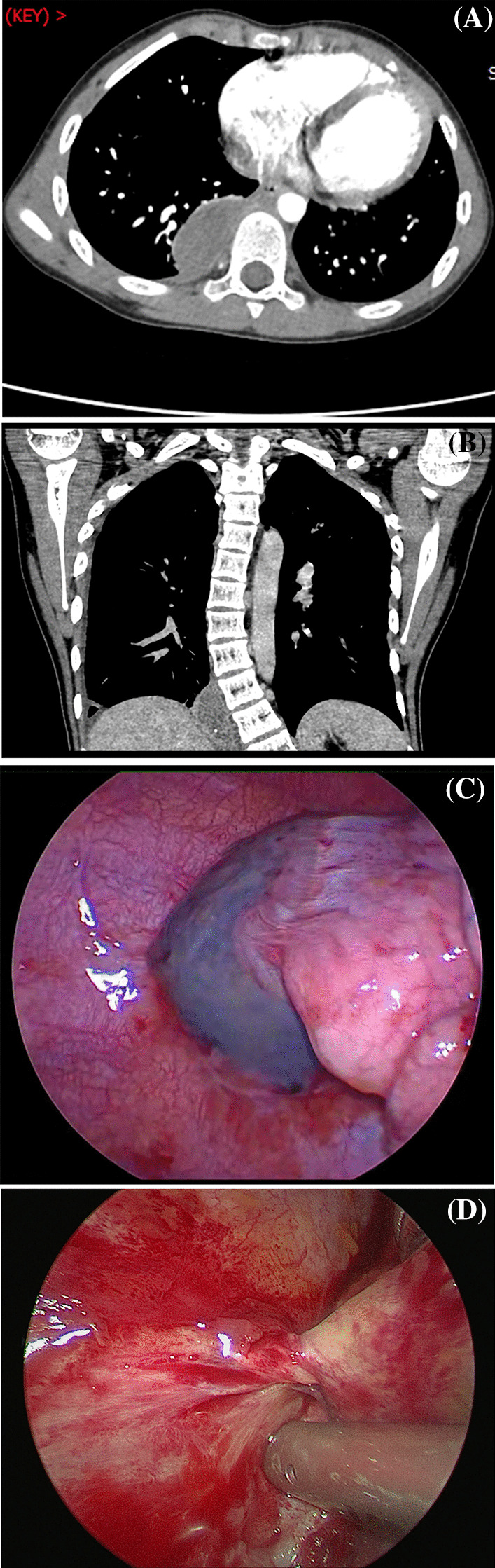
Axial Contrast-enhanced CT image of the chest **A** indicates that a well-bordered, soft-tissue density mass without enhancement in the right thoracic cavity, coronal CT image **B** shows a well-defined soft tissue mass between the diaphragm and the spine, video-assisted thoracoscopy **C** reveals a purplish-red mass with congestion and necrosis in the pleural cavity, and **D** shows a twisted vascular pedicle

Preoperative colour Doppler ultrasound and chest computed tomography (CT) examination revealed a well-bordered mass with soft-tissue density between the diaphragm and the lower lobe of the lung accompanied by a small amount of pleural effusion in 2 cases, medium pleural effusion in 3 cases and large pleural effusion in 1 case. On contrast-enhanced CT scans, the mass showed no obvious enhancement, the blood supply was only presented in 1 case, and no feeding artery was shown in the other 5 cases. On serial imaging, the pleural effusion and the mass diameter increased progressively in one child (Fig. 1).

A total of 3 patients were diagnosed as pulmonary sequestration or pulmonary sequestration with torsion by CT-scan or Colour Doppler ultrasound before surgery, and the remaining 3 cases were not clearly diagnosed by CT-scan or ultrasonography. One patient with imaging findings of pleural malignancy was finally diagnosed by thoracoscopy. A thoracoscope was used for exploration in one undiagnosed children. Pathological examination of the resected tissue specimens confirmed extralobar pulmonary sequestration with haemorrhagic infarction. Of the six cases with pulmonary sequestration, two were accompanied by myofibroblast proliferation.

### Treatment and prognosis

Eventually, all 6 children underwent thoracoscopic pulmonary sequestration resection. After entering the chest cavity, a dark red or purplish-red mass with intact capsule, congestion and necrosis was seen, accompanied by varying amounts of bloody pleural effusion in the pleural cavity. The feeding artery that originated from the intercostal artery in 3 cases and the descending aorta in 3 cases was observed intraoperatively. The ELS vascular pedicle was twisted several times at its origin. The vascular pedicles of all lesions were clipped with metal clips and then cut off.The median procedure time was 60 min (range, 60–120 min), and intraoperative haemorrhage ranged from 1 to 10 ml (median, 4 ml). The children did not have any intraoperative or postoperative complications, and their symptoms were relieved immediately after surgery. On postoperative days 2–6, they were discharged home uneventfully. There were no complications during a median follow-up of 4 months (range, 0.4–8.7 months).

## Discussion

Symptomatic ELS caused by pedicle torsion is extremely uncommon in clinical practice, and early diagnosis is challenging.It has been reported that only 1 case of vascular pedicle torsion occurred in 13 cases of ELS treated in a single centre from 2000 to 2009 [8]. In the past 14 years, a total of 32 cases of ELS were diagnosed in our hospital, of which only 6 cases were combined with torsion of the vascular pedicle, accounting for 18.75% of cases. To date, a total of 13 paediatric cases of ELS with torsion have been reported in the English literature [4–6, 8–17]. To the best of our knowledge, this is the largest number of cases reported in the literature. Among the 13 cases of ELS reported in the previous literature, including 9 males and 4 females, 12 cases were located on the left side and 1 case on the right side. The patients were between 4 and 13 years old. The left/right ELS ratio was 1:2 in our centre’s cases. The proportion of ELS on the right side was larger in our study, while previous literature found that ELS tended to occur on the left side [4–7], which may be one of the reasons for the low preoperative diagnosis rate. In our cases, one child was an adolescent, three were school age, and two were preschool age. The male to female ratio was 1:1. The age range in our study was similar to that reported in the literature, but the sex ratio differed. ELS is associated with other congenital anomalies, such as congenital diaphragmatic hernia, congenital pulmonary airway malformation or congenital heart disease. However, it is very interesting that none of the 6 children in our study had other congenital malformations.

Clinically, ELS with torsion shows no specific manifestations or symptoms, and it is difficult to diagnose. The literature showed that chest pain or discomfort was recorded as the main symptom among adult patients [18, 19], whereas abdominal pain was the primary clinical manifestation in children [5, 8–11, 13–16], followed by chest pain [6, 12–15]. Some children had fever [10, 15] and gastrointestinal symptoms such as vomiting [6, 9, 11–13].

Abdominal pain seems to be the primary symptom of ELS with torsion in children. 80% of ELS in children is located between the lower lobe of the lung and the diaphragm, typically in the left hemithorax [9]. The type of abdominal pain is likely similar to the pain experienced by patients with right lower lobe pneumonia [9], which makes it easy to misdiagnose [5]. The scope of the CT scan should include the lower thorax to the level of the pulmonary veins when evaluating acute abdominal pain to rule out the possibility of lung disease [14]. The cause of the abdominal pain may be due to local inflammation caused by ischaemic necrosis after torsion of the ELS vascular pedicle. The children’s symptoms disappeared immediately after removing the lesion [4, 12, 13]; however, antibiotic treatment could not have achieved this effect [13].

Imaging examinations, such as Doppler ultrasound, CT and magnetic resonance imaging (MRI), can play an important role in confirming the diagnosis. The discovery that the blood supply artery originates from the branch of a systemic artery rather than a pulmonary artery is strong evidence for the diagnosis of ELS [6, 14]. Unfortunately, torsion of the vascular pedicle hinders the visibility of the blood supply, resulting in unrecognizable blood supply vessels and atypical imaging findings [14]. Therefore, it is difficult for radiologists to diagnose ELS correctly before surgery. Additionally, there were atypical clinical symptoms in the children. These issues lead to higher rates of delayed diagnosis or misdiagnosis [9]. The preoperative diagnosis rate of ELS presenting with torsion is extremely low in childhood and adolescence. Among the 13 reported paediatric cases of torsed ELS in the literature, only 4 cases were diagnosed before the operation [4, 6, 10, 11]. In the early years, owing to a lack of awareness about the imaging characteristics of ELS with torsion among radiologists in our hospital, the first 3 cases were not identified before the procedure. After studying, analysing and summarizing the imaging characteristics of enhanced CT among radiologists, the last 3 cases were accurately identified before surgery. On contrast-enhanced CT scan, the main imaging signs of ELS with torsion are as follows: the mass has no obvious enhancement or only edge enhancement, and its feeding artery is unclear or not displayed accompanied by pleural effusion [5, 9–11, 14–16, 19]. Additionally, a rapid increase in pleural effusion or the mass size in a short period of time is considered to be one of the characteristic manifestations of acute torsion. Compared with CT, MRI seems to have more advantages in distinguishing ELS with pedicle torsion: lack of enhancement in the peripheral portion of the lesion with haemorrhaging within the mass, and vascular pedicle was well visualized [10].

The exact reason for torsion of ELS remains unclear. The literature suggests that activity or respiratory exertion may be the predisposing factor for vascular pedicle torsion; for example, some patients had performed vigorous activity (track competition, tennis) prior to the onset of illness [4]. Unlike the literature, reviewing the medical history of the children in our cases revealed that they did not have a history of similar vigorous activity before the onset. An effective treatment for ELS is surgery. Minimally invasive video-assisted thoracoscopic surgery is a safe and effective treatment for PS and has considerable long-term effects [20]. Therefore, thoracoscopy remains the preferred procedure for both the diagnosis and treatment of this disease, especially for bilateral lesions [7, 8, 13–16, 21].

## Conclusions

The ELS with torsion usually occurs in children or adolescents and has abdominal and/or chest pain as the primary symptom. Imaging examination shows a well-defined mass comprised of soft tissue between the lower lobe of the lung and the diaphragm. Contrast-enhanced CT shows that each mass had no enhancement or only marginal enhancement. The feeding vessel was not clearly displayed, and it was accompanied by varying amounts of pleural effusion. Video-assisted thoracoscopic surgical resection for ELS is associated with excellent prognosis.

## Data Availability

The data were presented in the main manuscript.

## Abbreviations

PS: Pulmonary sequestration
ELS: Extralobar pulmonary sequestration
ILS: Intralobar pulmonary sequestration
CT: Computed tomography
MRI: Magnetic resonance imaging
